# Medical Representation

**Published:** 1921-05-14

**Authors:** 


### The Hospital

The Workers' Newspaper of Administrative Medicine and Institutional Life. Administration. National Insurance and Health.

No. 1823, Vol. LXX. SATURDAY, MAY 14, 1921. PRICE TWOPENCE

### MEDICAL REPRESENTATION.

Those of our readers interested in evolution of an organisation by which free and representative discussion of professional problems can be obtained will not be surprised that certain divisions of the British Medical Association have already registered their objection to the project of affiliating to the B.M.A., certain societies in allied sciences. To us it seems perfectly clear that a medical association, however satisfied it may at the moment be with its own representative strength, had far better, if it wishes to carry confidence as well as the greatest membership among the doctors, devote its main energies to the medical profession alone. Any scheme whereby the B.M.A. should also become a federating body in the widest professional sense can only, we think, result in diminishing its actual attention to the problems of the doctor himself.

It is therefore with interest that one reads from a meeting of the Reigate Division of the B.M.A., a discussion on these proposals for federation. As we have previously pointed out, if a Federation is required—and the principle is undoubtedly the only one by which the collective professional voice can be heard—then the organisation is already in existence as the Federation of Allied and Medical Societies. At the above meeting the Secretary of this body, Dr. N. Howard Mummery, when asked to define its position in relation to the B.M.A., put the case perfectly clearly. Only those who choose to do so make it out as one of antagonism. The Association itself, represented by Sir Jenner Verrall, was one of the bodies which met a large number of others in 1918, when it was voted that such a body as the Federation should be set up. Later the B.M.A. put forward a claim to form and control the new body itself. The majority of the societies disagreeing, however, they formed the existing Federation, hoping that the B.M.A. would see its way to come in. Thus the Federation of Allied and Medical Societies is supplementary, and not in opposition, to the present organisation of the Association. The former represents practically all the allied professions, and if the chief medical body will only accept the hand of friendship, which is still extended, a united profession in the widest sense is at once available. In the interests of efficiency, as well as true representation, we hope that medical men will see the obvious superiority of this last procedure.

